# Predicting Cancer Patient Survival with Gene Expression Data

**DOI:** 10.1371/journal.pbio.0020118

**Published:** 2004-04-13

Cancer specialists often talk about cancer as an umbrella term for over 200 different diseases, each having unique characteristics. But even these categories are too broad, as the same type of cancer can take very different paths in different people. It's not uncommon, for example, for a tumor to grow aggressively in one patient and stabilize or regress in another, even though their tumors are indistinguishable and are treated in the same way. Researchers have traditionally diagnosed and treated cancer based on microscopic analysis of cell size and shape, a method that's especially difficult for very closely related cancers, such as non-Hodgkin's lymphoma, which has 20 subtypes. As scientists learn more about the molecular alterations in cancer, they're beginning to establish cancer subtypes based on the underlying molecular footprint of a tumor. Four years ago, DNA microarray analysis revealed that the most common subtype of non-Hodgkin's lymphoma is in fact two separate diseases. Though the tumor cells of both cancers appear large and diffusely dispersed in a tissue sample under a microscope, each has a distinct genetic profile, possibly explaining why only 40% of patients with this subtype respond to the standard chemotherapy treatment.[Fig pbio-0020118-g001]

**Figure pbio-0020118-g001:**
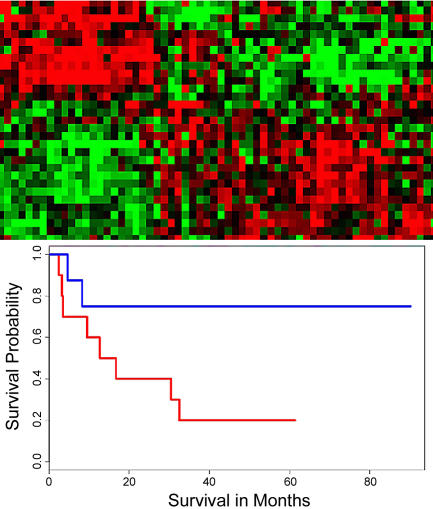
Selecting expression profiles that can predict cancer outcome

Such molecular pathology has led to the discovery of subtypes of several different tumor types and has successfully identified patients with different survival times. But such correlations work best when cancer subtypes based on genetic profiles are already known. If you know that different subtypes exist and which patients belong to which subtype, then you can build a statistical model to diagnose such cancers in future patients. But in most situations, clinicians don't know either of these variables—or even whether such a subtype exists—information that is crucial to developing effective diagnostic and treatment protocols. Statistical methods to identify such subtypes exist, but they can generate classifications that lack clinical relevance. Now Eric Bair and Robert Tibshirani describe a procedure that combines both gene expression data and the patients' clinical history to identify biologically significant cancer subtypes and show that this method is a powerful predictor of patient survival.

Their approach uses clinical data to identify a list of genes that correspond to a particular clinical factor—such as survival time, tumor stage, or metastasis—in tandem with statistical analysis to look for additional patterns in the data to identify clinically relevant subsets of genes. In many retrospective studies, patient survival time is known, even though tumor subtypes are not; Bair and Tibshirani used that survival data to guide their analysis of the microarray data. They calculated the correlation of each gene in the microarray data with patient survival to generate a list of “significant” genes and then used these genes to identify tumor subtypes. Creating a list of candidate genes based on clinical data, the authors explain, reduces the chances of including genes unrelated to survival, increasing the probability of identifying gene clusters with clinical and thus predictive significance. Such “indicator gene lists” could identify subgroups of patients with similar gene expression profiles. The lists of subgroups, based on gene expression profiles and clinical outcomes of previous patients, could be used to assign future patients to the appropriate subgroup.

An important goal of microarray research is to identify genetic profiles that can predict the risk of tumor metastasis. Being able to distinguish the subtle differences in cancer subtype will help doctors assess a patient's risk profile and to prescribe a course of treatment tailored to that profile. A patient with a particularly aggressive tumor, for example, would be a candidate for aggressive treatment, while a patient whose cancer seems unlikely to metastasize could be spared the debilitating side effects of aggressive anticancer therapies. By providing a method to cull the thousands of genes generated by a microarray to those most likely to have clinical relevance, Bair and Tibshirani have created a powerful tool to identify new cancer subtypes, predict expected patient survival, and, in some cases, help suggest the most appropriate course of treatment.

